# Clean and sustainable environment problems in forested areas related to recreational activities: case of Lithuania and Turkey

**DOI:** 10.3389/fspor.2024.1224932

**Published:** 2024-02-23

**Authors:** Ahmet Atalay, Dalia Perkumiene, Marius Aleinikovas, Mindaugas Škėma

**Affiliations:** ^1^ School of Physical Education and Sport, Ardahan University, Ardahan, Turkey; ^2^ Department of Business and Rural Development Management, Faculty of Bio Economy Development, Agriculture Academy, Vytautas Magnus University, Kaunas, Lithuania; ^3^ Institute of Forestry, Lithuanian Research Centre for Agriculture and Forestry, Kaunas, Lithuania

**Keywords:** forest, ecosystem, recreation, sustainability, clean environment

## Abstract

**Introduction:**

With the acceleration of social life, people's interest and demand for forestry recreation activities is increasing. However, with this increase, it is inevitable that negative environmental effects will occur. Particularly mass participation poses an important risk for environmental sustainability. In this context, the aim of this study is to determine the recreational activities organized in forest areas in Turkey and Lithuania, the environmental effects of these activities and the precautions to be taken.

**Methods:**

In Turkey and Lithuania, interviews were conducted to determine the attitudes of experts involved in recreational activity processes towards a clean environment and environmental sustainability. A semi-structured interview form was used in the interviews with forest operators and other experts. The sample group of the research consists of 17 experts from Turkey and Lithuania.

**Results:**

According to the results of the research, recreational activities are organized in forest areas in both countries, but the most important problem related to these activities is waste production. In addition, there is also damage to the natural environment. Although there are legal regulations in both countries, there are no definite results in solving environmental problems.

**Conclusions:**

It can be said that necessary measures such as raising awareness of people and ecological education should be taken in order to ensure the right of individuals to live in a safe and clean environment and at the same time to ensure sustainability in forest areas. as the improvement of legal regulation.

## 1 Introduction

Urban green infrastructure provides a range of experiences for people and various health benefits that support human well-being (1). Visiting natural environments in nature and being a part of outdoor activities have positive effects on human health. It also contributes to the awareness of the development of social connections and the protection of natural and cultural heritage (2, 3). However, it also contributes to sustainability while ensuring the development of individuals and societies (4). It should be noted that properly organized recreation activities make a person's life fulfilling and happy, regardless of a person's work or position in society. It is valuable, with the help of recreation a person can satisfy many basic physical and mental needs and at the same time strengthen his health (5, 6).

However, when we talk about recreation, it is not enough to say that it is only an activity of leisure. Recreational activities, which play an important role in social life in terms of health and social interoperability, have fundamental and distinctive characteristics. Recreation gives a feeling of freedom, it is done in free time, gives pleasure and joy, is an activity against laziness. Recreation provides instant satisfaction and is immediately in the activity and allows the routine to change, the choice of recreational activities is based on voluntary basis, the meaning of recreation may differ according to the participant, recreation is individual, for someone else. Recreation as leisure activities are expected to bring individual and social benefits and be socially appropriate (7). Recreation activities also have an important sociological role in coping with times of social crisis or tragedy (8). It is seen that the participation of recreation activities increases depending on this social function. However, each individual human activity generates waste, and as the demand for quality of life increases, the amount of waste tends to increase (9). In conjunction with these increased demands, human-induced disturbances and public parks, forests, wilderness and public parks It is known that adverse changes occur in the environmental conditions of private lands (10).

As for recreational activities, we must discuss these activities in forest areas, including protected forest areas. Protected forests are geographical areas defined and managed by legal or other effective measures in order to ensure the long-term preservation of nature with ecosystem functions and cultural values (11). The use of these areas for recreational activities and the organization of various activities are increasing. While the effects of recreational activities on human health are known, the damage to the natural structure and forest areas can be considered as a threat. Recreational activities with intense participation have many potential negative effects such as water consumption, high energy consumption, emission production, noise, garbage and disruption of ecosystems (12, 13). In addition, outdoor recreational activities such as hiking, swimming and fishing can all be affected by plastic pollution. Plastic pollution can create major health problems (14).

The world is undergoing major changes in the quality of the environment due to human activities. Due to intensive industrial development, urbanization of large cities, as well as inappropriate use of forests for economic and recreational activities (15). The rapid pace of life has led to several global problems and challenges that are vital to human beings, one of the most pressing of which is environmental pollution and its damage to the environment, which poses a threat to human health and even life. Recreational activities organized in forests and other wilderness and natural open spaces offer many individual and societal benefits and opportunities. It is also very important for the ecosystem. However, the attitudes and behaviors of visitors can cause damage to the forest and nature (16). Selfish use of the environment, including forests and the goods they provide, without making efforts to restore the former state of the environment and its components, can force humanity to an ecological catastrophe. Understanding and acknowledging the fact that the process of restoring the environment is a long and complex process, and in certain cases, when irreversible environmental changes occur because of human activities, it is generally impossible, although various measures are generally taken to preserve the environment around us. The surrounding environment is extremely important not only for us, but also for future generations (17). Participation in recreational activities organized in woodlands and protected areas has negative impacts on the natural environment. This has a negative impact on the balance between the natural environment and recreation activities. Therefore, a worrying process of environmental degradation is discussed (16). It is inevitable to improve the attitudes and behaviors of individuals and to increase environmental awareness and consciousness in order to address these increasing concerns related to human activities (18).

The research is limited to forested areas located in Burdur city in Turkey and forested areas located in Kaunas city in Lithuania. This research was limited to one forest area open to recreation activities in Turkey and Lithuania. Firstly, the fact that the researchers were from Turkey and Lithuania was decisive in the country limitation. However, Lithuania is a European Union country. Turkey is in the process of membership to the European Union. In this context, the understanding that two countries with administrative and political differences may have similarities and differences on a common subject reveals the limitation of the research. It is aimed that the research findings will be an inspiration for future research and to be used as an opportunity for studies involving more than one country city. Therefore, it is important to timely identify threats to the environment and prevent damage. A clean and safe environment is one of the most important goals of most states and all citizens. Ensuring human rights to a safe and healthy natural environment and environmental protection towards sustainability are among the most relevant and popular socio-economic as well as legal-political issues. With the acceleration of social life, people's interest and demand for forestry recreation activities is increasing. However, with this increase, it is inevitable that negative environmental effects will occur. Particularly mass participation poses an important risk for environmental sustainability. In this context, the aim of this study is to determine the recreational activities organized in forest areas in Turkey and Lithuania, the environmental effects of these activities and the precautions to be taken.

Focusing on forest recreation activities and negative environmental impacts, this study aims to contribute to our understanding of the myriad benefits, impacts and complexities associated with human interactions in natural environments. It contributes to the existing literature by exploring emerging trends in this process and providing actionable insights for policy makers, educators and practitioners in the field of nature-based recreation. This research helps us to make more informed decisions about how we can capitalise on the positive aspects of our relationship with nature while protecting and conserving our natural environments.

## 2 Background

### 2.1 The concept of recreation and recreation activities in the forest

Today, factors that cause mental and physical negative effects such as the increase in urban life, heavy traffic, work pace, and stress have led people to participate in activities that will entertain them in their spare time and provide physical and spiritual satisfaction. For this reason, the phenomenon of leisure time has gained importance today and has revealed the concept of recreation (19). Recreation: It is necessary for people to get away from their daily lives, to rest physically and mentally, and to spend their free time on personal development. In free time, it includes all activities done to get rid of the boring, disciplined, and monotonous effects of daily life and to relax (20). In today's societies, the importance of recreational activities is increasing in order to protect physical, social and mental health. However, due to the negative conditions in the cities and the insufficient recreational resources, the people living in the city tend to the recreation areas outside the city (21).

Recreational areas, which are planned to meet the needs of people such as sports, aesthetics, health, and rest, can be in a natural environment as well as in urban forests created around the city. In this context, the trends towards recreational use of forest areas have increased in recent years (22). Recreational activities in forest areas have gained increasing importance in recent years (23). Recreation is the most important of the non-marketed services provided by the forest ecosystem. Many environmental assessment studies have been conducted to determine the value of in-forest recreation in terms of socio-economic factors (24–27). Forests near cities are recognized as providing recreational opportunities for people (28, 29). Recreation activities in natural areas such as forests are a common phenomenon today. Demands for activities such as cycling, skiing, hiking, and picnicking in the beautiful scenery increase the number of visitors to these places (30, 31).

With the acceleration of social life and the intensification of urban life, the importance of recreational activities is gradually increasing. The dissemination of these activities is inevitable in terms of protecting and improving the physical and mental health of people. Spending time in natural environments such as forests has been found to positively affect people's mental and physical health (32). For this, activities are planned according to the wishes and needs of the people. These activities are increasingly concentrated in open space and forest areas. Recreational areas, which are planned to meet the needs of people such as sports, aesthetics, health, and rest, can be in a natural environment as well as in urban forests created around the city. In addition, research suggests that the nature experience itself is more important than the effects of physical activity and social interaction (33). Therefore, increasing outdoor recreation can be considered beneficial both at the individual level and for society as a whole (34). Erosion and natural life pollution occur due to visitors in the recreation areas within the forest. According to various studies conducted by scientists, many negative effects such as garbage, noise, traffic, commercial exploitation, and fire cause destruction in these areas found with traffic should point more related sources there related with traffic, noise etc.) (30, 35). Therefore, when we are talking about the benefits and advantages of recreation, and highlighting these problems and issues, we must also discuss environmental damage and the public's right to a safe and clean environment seeking balance and sustainability both in nature and in all human activities, as well as in recreation.

### 2.2 The concept of environmental damage

In the legal literature, environmental damage is understood as a separate and special type of damage that cannot be unconditionally assigned to either property or non-property damage. This type of damage is dual in nature: it has both material and non-material damage. For example, if a forest is illegally cut down, the property interests of the forest owner are violated. At the same time, damage is done to the non-property interests of the forest owner and society, as the forest loses its environmental, natural, and other functions. The legal literature emphasizes that the forest is a special property object (36): (1) The forest is an object of private or state property, therefore, causing damage to the forest causes damage to the interests of forest managers, owners or users; (2) The forest is an environmental component that performs environmental (ecological) and social functions (protects the stability of the landscape, improves the quality of the environment) (37).

Recently, when about 54% of the world's population lives in cities, where is faced with heavy traffic and the environment is polluted by factories and thus is caused enormous damage to the environment. Air pollution is particularly harmful to human health. Damage for the environment, and at the same time to human health, is also caused by indoor pollution due to cooking with dirty fuel or inefficient technologies (especially in India and China), as well as burning candles and wood or smoking indoors (38). Environmental damage is occurring on a global scale. Also, scientists note the local factors of environmental destruction (39, 40), when they are talking about environmental damage, they discuss remediation, which is the repair of damage caused by past human activity or natural disaster clean up technology (41). According to the authors, environmental damage can be reduced by modifying or adding pollution reduction measures to the installed production plant. The equations should be inserted in editable format from the equation editor.

In terms of forest damage (42), highlight the damage caused by the mountain pine beetle (MPB) (Dendroctonus ponderosae) to pine trees. Though these beetles are native from the US forests they play an important ecological role, as they can cause significant tree death. Climate change has increased these harmful effects. This increase is associated with changes of temperature and increased water stress, which create favourable conditions for the beetles to survive and grow in the trees (42).

Environmental damage is defined in the Law of the Republic of Lithuania on Environmental Protection as “directly or indirectly, a negative change in the environment or its elements, including protected territories, landscape, biological diversity, or deterioration of their functions, available properties, beneficial to the environment or people (society)”. Article 32 of this law recognizes that damage to the environment has been caused if there is a direct or indirect negative impact on environ-mental elements: surface and underground water, the surface of the earth, forests, landscape, protected areas, the state of biodiversity (their functions), when environmental protection requirements are violated (43). According to the scholars, in order to understand the impact of the distribution of environmental damage, it is necessary to distinguish between cases where populations are affected by different levels of environmental goods and cases where gradual environ-mental change can have different effects (44). Danger to human life is also caused when the land, water, and ambient air are polluted with harmful chemical and radioactive substances that exceed the permissible norms. Therefore, environmental protection laws limit or generally prohibit any economic activity related to the release of harmful pollutants into the environment and indirectly ensure the public's right to a safe environment (45–47).

The responsibility for environmental damage lies with “operators”, a broad category of persons engaged in economic activities. They are established based on strict liability, although there are certain activities which are not exempted and certain defences, some of which are optional in the sense that their implementation under national law is left to the discretion of each Member State (48).

### 2.3 The public's right to a clean environment

It should be noted that both in scientific literature and in legal documents, the most used concepts are the right to a healthy and clean environment and the right to a healthy and safe environment. From a human rights point of view, the right to a healthy and quality environment is a fundamental right (49). Lithuanian court practice and legal scientific research in the analysed area are in the formative stage, but it can be said that the situation is improving, with the increase of judicial practice and scientific works on this issue. Recently, the public right to a safe environment has not been directly formulated in the Constitution and laws of the Republic of Lithuania (43). In Turkey, the Constitution of the Republic of Turkey, Article 59 states that “Everyone has the right to live in a healthy and balanced environment. It is the duty of the State and citizens to improve the environment, protect environmental health and prevent environmental pollution” (50). In Turkey, the right of people to live in a clean environment and to access this environment is guaranteed by the constitution. In this way, a healthy environment and human life were guaranteed by laws. The Law on Environmental Protection established the right of the citizens of the Republic of Lithuania to a healthy and safe environment, but that right somehow disappeared in subsequent editions of this law. The constitutions of other European Union states: Bulgaria, the Czech Republic, Spain, Latvia, Portugal (51) and Finland enshrine the right to a healthy and clean environment, i.e., this right has been given the status of a constitutional right. However, the specific content of this right is largely undisclosed in the constitutions of the mentioned states, and there is no clear answer to this question in the legal doctrine (52).

Although the public right to a safe environment is not clearly stated, it cannot be said that it does not exist at all. The mere fact that the duty of the state to take care of the protection of the natural environment and particularly valuable areas, enshrined in Article 54 of the Lithuanian Constitution, presupposes the public's right to live in a safe environment (43). The Rio Declaration on Environment and Development also emphasizes the human right “to a healthy and fulfilling life in harmony with nature” (53). In the report “Environment and human rights”, based on Principle 1 of the Stockholm Declaration “On the Human Environment”, recommends member states to recognize the human right to a healthy, suitable environment (54). The task formulated in the Treaty on the Functioning of the European Union is a high level of environmental protection and improvement of its quality. This means that a high level of environmental protection must be maintained by stopping any activity that has already started that is damaging the environment or, although it does not have a negative effect on the environment but may do so in the future. The environmental objectives of the Community are listed in Article 191, Part 1 of the Treaty (55).

The public's right to a safe and clean environment is directly related to the human right to health protection which is enshrined in the primary law sources of the European Union. This task is formulated in the Treaty on the Functioning of the European Union is a high level of environmental protection and improvement of its quality. This means that a high level of environmental protection must be maintained by stopping any activity that has already started that is damaging the environment or, although it does not have a negative effect on the environment, but may do so in the future (56, 57).

## 3 Methods

### 3.1 Methodologic structure

**Figure 1:**
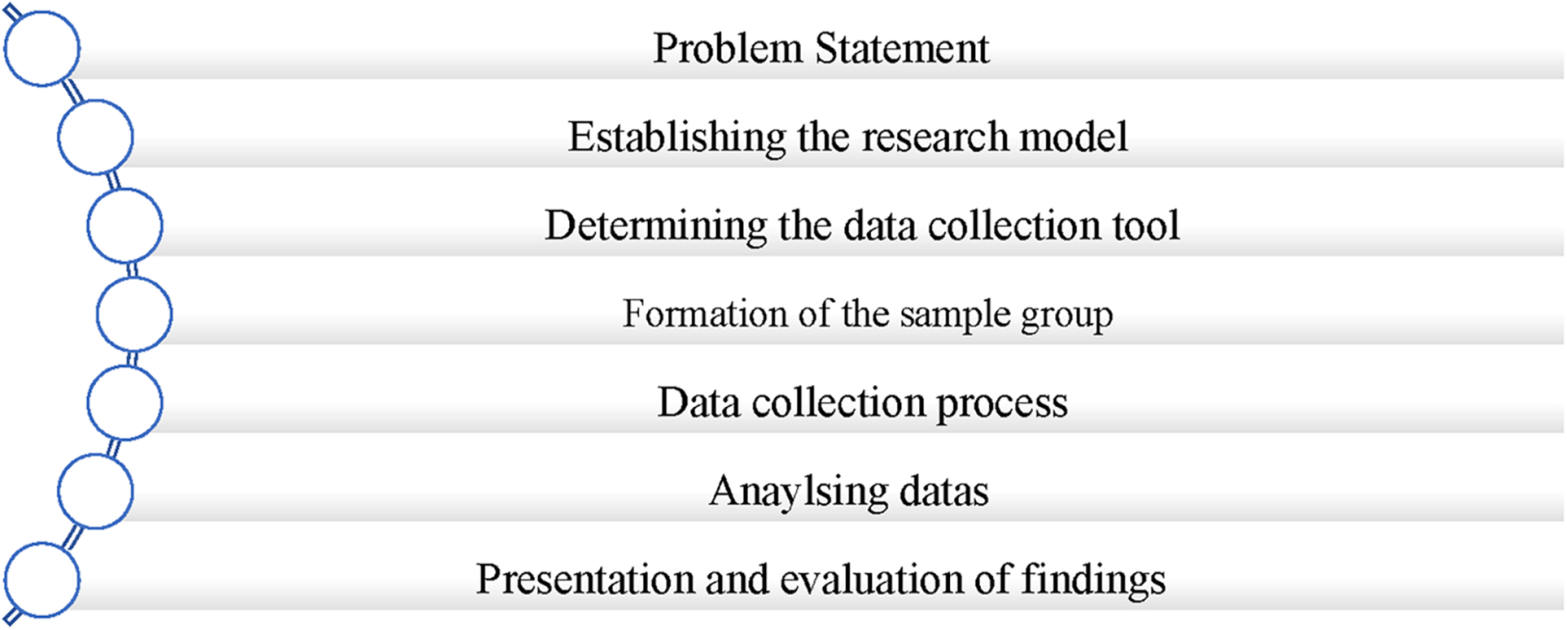
Research process.

In this research, the case study pattern was used within the scope of qualitative research methods, which are frequently used in social sciences in recent years. The qualitative dimension of the research consists of two steps. In the first step, the existing literature was examined in order to reveal the relationship between forestry recreation activities and environmental sustainability and to specify the conceptual framework of these areas. In the second step, interviews were conducted to determine the views of experts open to recreational activities in Turkey and Lithuania on clean environment and environmental sustainability. In this context, a plan for the course of the study was created and the research process was visualized in Figure 1 below:

### 3.2 Sample group

Purposive sampling method was used in the selection of the participants for the interviews. Purposeful sampling is a method that allows us to provide in-depth information by identifying information-rich people, institutions and situations in accordance with the purpose of the research and it is envisaged to limit the interview groups to a maximum of 20 people in order to have in-depth information (58, 59). It is convenient to have a smaller number of research participants so that each case can be studied more deeply, and such participants are needed, whose special qualities provide the best reflection and knowledge about the research phenomenon (60–62). The sample group consists of people who are experts and competent in environmental policies, especially forest area managers where recreational activities are organized. Therefore, it is appropriate to work with a smaller number of participants in order to conduct in-depth research in a specific field, and they are expected to provide the most accurate and accurate information about the research topic thanks to their special qualifications (61).

The sample group of the research consists of 17 interviewers in total from Turkey and Lithuania. 8 of them are managers responsible for forest areas open to recreational activities. It is based on the determination of the responsible persons as experts in the monitoring and follow-up of all activities organized in the forest area. 4 of them are academicians related to the research subject. It is aimed to include researchers who have produced studies that deal with the environmental sustainability problem related to the research subject, as experts. Five of them are experts in sports and recreation sciences. Finally, experts who organize different recreational activities in forest areas and who have experience in theory and practice, and who carry out sports and recreation research, formed the last group of the working group. For the mentioned interviews, a total of 17 experts, nine from Turkey and eight from Lithuania, were determined for the sample group. Although the experts in the research group were kept anonymous, they took part in the presentation of the research findings and coded in direct quotations. Forestry managers in Turkey were coded as TFM (forest managers in Turkey), and managers in Lithuania were coded as LFM forest managers in Lithuania. Experts conducting environmental sustainability research in Turkey were coded as TE (experts in Turkey), and managers in Lithuania were coded as LE (experts in Lithuania). Finally, sports and recreation experts in Turkey are coded as TSE (sport science expert in Turkey), and managers in Lithuania are coded as LSE (sport science expert in Lithuania). Demographic information about the experts participating in the research is presented in Table 1.

**Table 1:** Demographic information about the experts.

Experts	Age	Gender	Education	Experience
Turkey				
TFM1	44	Male	Bachelor's degree	18 years
TFM2	38	Female	Master	11 years
TFM3	49	Male	Bachelor's degree	21 years
TFM4	28	Male	Bachelor's degree	4 years
TE1	43	Female	Ph.D.	18 years
TE2	51	Female	Ph.D.	24 years
TSE1	37	Male	Master	7 years
TSE2	39	Female	Master	13 years
TSE3	39	Male	Bachelor's degree	13 years
Lithuania				
LFM1	50	Male	Bachelor's degree	24 years
LFM2	47	Female	Master	17 years
LFM3	34	Female	Bachelor's degree	6 years
LFM4	41	Female	Master	14 years
LE1	53	Male	Ph.D.	24 years
LE2	46	Male	Master	13 years
LSE1	33	Female	Master	10 years
LSE2	39	Male	Master	13 years

### 3.3 Data collection tool

A semi-structured interview form was used in interviews with forestry managers and other experts. For the validity and reliability of the semi-structured interview form used in the research, the volunteering of the participants will be taken as a basis. Semi-structured interviews are a valuable tool for gathering qualitative data in social science research. By providing a balance between structure and flexibility, they allow researchers to explore participants' experiences, opinions, beliefs, and attitudes in a detailed and nuanced way. In this form, in addition to the questions prepared beforehand in the interviews, there is the freedom to ask additional questions that are not written in the interview forms, if needed, depending on the course of the interview. This feature of the interview gives flexibility to the research (59: 128). In addition, according to Karasar (63), survey questions are determined in advance in semi-structured interviews and data is tried to be collected with these questions. Such interviews combine both fixed choice answering and being able to go in-depth in the relevant field (64: 234). In addition, semi-structured interview is an interview technique prepared with the aim of revealing the participants' own thoughts and solving the problems that are required to be clarified (65). Each researcher is expected to carefully test the validity and reliability of the data collection tools and research model they use and to report the results to the reader (59: 255). The following suggestions will be considered for the validity and reliability of the semi-structured interview form to be used in the research:

• Since the questions in the interview form are in English, they will be checked by language experts and will be finalized after corrections.
• In the interviews, the interview process will be explained in detail and the volunteerism of the participants will be taken as a basis.
• The same questions will be asked in the same words and in the same way to each interviewee.
• All raw data of the study will be stored so that it can be reviewed by others (66).

Semi-structured interview is an interview technique prepared with the aim of revealing the participants' own thoughts and solving the problems that are required to be clarified (65). A semi-structured interview is a type of interview form used in qualitative research. In this form of interview, the interviewer has a set of pre-determined questions but can also ask follow-up questions based on the participant's responses. This type of interview form allows for both flexibility and structure, providing the researcher with the opportunity to gather rich and detailed data while still maintaining a degree of control over the conversation (67). The questions in the semi-structured interview form were prepared in accordance with the purpose of the research are given below:

1. What recreational activities are organized in this area?
2. What problems and challenges arise when organizing recreational activities in forest areas?
3. What legislation must be considered when protecting the environment?
4. What are the measures taken for environmental protection concerns to organized events?
5. How can be protected clean and safe environment and human rights to a clean and safe environment in forest areas in organizing recreational activities seeking sustainability?

For the validity and reliability of the research questions: The same questions were asked in the same words and in the same way to each interviewee. The questions are purpose-built. The statements in the interview form were confirmed. While preparing the interview form, the relevant literature was examined and expert opinions were taken (68, 69). During the interviews, the participants included in the study group were given preliminary information about the study, their appointment requests regarding the meeting date and time were sent, the interviews were recorded and then transcribed.

### 3.4 Analysis of data

In the first step of the qualitative dimension of the research, document analysis method was used within the case study design. Document analysis is a method based on the detailed, meticulous, and systematic classification and analysis of written documents (70). Therefore, it provides the opportunity to analyse and interpret the existing data to make a certain meaning with document analysis and to reveal an understanding of the subject (71). Systematic review and meta-analysis methods are often preferred as part of a qualitative study. With this method, it is essential to present evidence on the topic under investigation by analyzing the existing literature in depth. Afterwards, evaluations can be made with a critical approach (72). Systematic review and meta-analysis processes aim to eliminate uncertainties and answer problems with current literature (73). In this study, a comprehensive literature analysis and evaluations are presented based on the PRISMA Preferred reporting items for systematic reviews and meta analyses (PRISMA) statement. In this study prepared in 2023, it has been tried to utilize the current literature as much as possible. Keywords were used in determining the scope of the literature. The keywords used in determining the literature addressed are as follows: “clean environment”, “recreation activity”, “forest area” “sustainable environment”, “recreation and environmental sustainability”. For a comprehensive literature assessment, a thorough screening is inevitable. For this, multiple and international databases were used. Searches through only one database reveal only a fraction of the existing literature. The use of multiple databases was crucial for a sound literature review (74). In order to access a large number of data, more than one database was used with the most appropriate keywords. These are international databases such as Scopus, Web of Science, EBSCO, ResearchGate etc. In the second step of the qualitative dimension, it is aimed to descriptively analyse the data obtained from the interviews with the help of the Nvivo 12 package program and to perform content analysis. In this process, the opinions of the participants will be conveyed directly (75). The answers obtained for each question posed during the data analysis process were summarized and visualized in tables. The evaluation and interpretation of the findings obtained in qualitative studies is part of the effort to establish meaningful relationships and is very important (76). However, direct quotation of participant opinions rather than quantification of the findings ensures that the thoughts of the individuals are reflected as they are (77).

## 4 Results

In this part of the research, the findings regarding the recreational activities organized in forest areas in Turkey and Lithuania. The environmental effects of these activities and the measures to be taken were included. The answers given by the participants to the questions in the interviews conducted through the semi-structured interview form compiled by the researchers is presented in the table below. However, to obtain detailed information, the opinions and thoughts of the interviewees were directly quoted and presented in accordance with the principle of clarity of data sharing (refer Table 2).

**Table 2:** Experts’ opinions on organized recreational activities.

Organized recreational activities in forest areas	
Experts in Tukey	Walking, Running, Biking
Experts in Lithuania	Camping, Forest Therapy, Educational Activities, Children's Camps, Kayaking, Horse Riding, Fishing, Nordic Walking, Cycling, Bird Watching.

The analysis results of the answers given to the question of which recreational activities are organized in forest areas open to recreation, shows that different activities and organizations are organized in Lithuania compared to Turkey. It is understood that the activities organized in Turkey are limited to running, walking and cycling (Experts in Turkey). The statements of the experts from Turkey who participated in the research are as follows:

*“Especially on weekends, events and organizations are organized by the youth and sports provincial directorate. These are running and walking races, biking, open to all ages” (TFM1). “We mostly organize running and walking activities. Such activities can be easier in terms of reaching more participants without the need for equipment” (TSE2). “I think that the most suitable activities for the natural environment and forested areas can be nature walks and cycling. I have participated in a few of them and I continue to participate. The activities I know are related to walking, running and cycling” (TE2).*

In Lithuania, in addition to walking, running and cycling activities, fishing, bird watching, horse riding, training camps, etc. are also organized (Experts in Lithuania). The statements of the experts from Lithuania who participated in the research are as follows:

*“Educational trails, excursions, recreational services for visitors, educational activities, events in nature, children's camps, kayaking, horse riding, fishing, cycling, bird watching are available” (LFM2). “From time to time there are horse riding activities that I also participate in. I think that spending time with nature and animals is very beneficial for human health” (LE2). “We organized a bike race once and the turnout was much higher than we expected. Another colleague of mine contacted the relevant institutions and organized a bird watching event” (LSE2).*

In line with the answers given to the question about the problems that emerged during the activities organized, it can be said that the activities organized in the forest area basically caused the problem of litter. The main problem that came to the fore in the interviews with the participants from both countries was the littering of garbage (Table 3).

**Table 3:** Experts’ opinions on problems and challenges arise when organizing recreational activities in forest areas.

Problems and challenges arise when organizing recreational activities in forest areas.	
Experts in Tukey	Garbage, Waste.
Experts in Lithuania	Garbage, broken and damaged recreational tools, hunting, water pollution, littering, illegal logging, felling of trees, poaching, illegal fishing and illegal means, damage to the forest floor, riding ATVs and motorcycles, large flows of people, illegal camping

*“Rubbish. In a word, garbage. Our people just leave their garbage and go. Especially plastic water bottles. Plastic is the most harmful substance in nature and does not disappear easily. All I can say for myself is garbage” (TFM2, TE1). Our most important problem is that participants throw their rubbish around irregularly during the events we organize. I constantly warn the participants about this issue, but we still cannot prevent rubbish from being left in nature. I think the most basic problem is the intensive production of rubbish” (TSE1). “Rubbish, rubbish, rubbish. People bring food with them when they come for a walk or any other activity. After consuming them, they can throw the packaging around randomly” (LFM3).*

In addition, it is seen that different problems arise in Lithuania due to the organization of different activities than in Turkey. It is understood that water pollution, illegal fishing and camping activities are carried out. In addition, it is understood that recreation tools and equipment are also damaged in Lithuania and the participants are insensitive to this issue

*“Water pollution, littering, broken and damaged recreational facilities, illegal logging, felling of trees, camping in unauthorized places, poaching, illegal fishing and illegal means, damage to the forest floor, riding ATVs and motor-cycles, large flows of people, illegal camping (LE2)”. “The waste and garbage left by vacationers, although recently, with the installation of more trash cans in the resorts and the increasing awareness of people, this problem is decreasing (LSE2)”. “The question arises as to who bears the responsibility for damage due to inappropriate exposure or neglect of recreational facilities. Another big problem is littering (LFM1)”.*

The reviewers' answers to the question regarding the legal regulation of the protection of forest areas revealed that the protection of forest areas open for recreation is legally regulated in both Turkey and Lithuania. Accordingly, regarding the protection of forest areas in Turkey, “Forest Parks Regulation”, “Forest Law”, “Forest Parks Implementation Communiqué”. In Lithuania “Law on Forests”, “Law on Protected Areas”, “Territorial Planning Law of the Republic of Lithuania “The Land Law of the Republic of Lithuania”, “Law on Environmental Protection” (Table 4).

**Table 4:** Experts’ opinions on legislation must be taken into account when protecting the environment.

Legislation must be taken into account when protecting the environment.	
Experts in Turkey	“Forest Parks Regulation”, “Forest Parks Implementation Communiqué”, “Law on Forest”
Experts in Lithuania	Law on Protected Areas”, “Law on Environmental Protection”, Law on “Forests of the Republic of Lithuania”, “Tourism Law”, “Territorial Planning Law”, “The Land Law”, “Law on Special Land Use Conditions”.

*“Forest Law”, “Forest Parks Regulation” and “Forest Parks Implementation Communiqué”. The forest law is about the protection of forests in Turkey. However, the other two regulations are more concerned with public woodlands (TFM1, TE2, TSE3)”. “Law on Protected Areas”, “Law on Environmental Protection” (LE2)”. “The main documents for the planning of recreational areas, the decisions of which depend on the needs, development goals, and characteristics of the territory” (LFM3).*

The results of experts answers analysis to the question regarding the measures taken during recreational activities are similar. Accordingly, it is seen that similar measures have been taken in both Turkey and Lithuania, such as increasing training and awareness, increasing more employees and volunteering activities (Table 5).

**Table 5:** Experts’ opinions on the measures taken for environmental protection concerns to organized events.

The measures taken for environmental protection concerns to organized events.	
Experts in Turkey	Education and awareness, preventive posters, putting more dustbins, comprehensive laws and regulations, more staff.
Experts in Lithuania	education and awareness, more staff, volunteer groups, traffic regulation, cognitive educational activities, comprehensive laws and regulations, cooperation warning signs.

*“We prepare extra information notes on the days when recreational activities are held. These notes contain information about the environment in which they live. In this way, we aim to make people pay a little more attention” (TFM1). Organizations organizing the event also support us in this regard. For example, after the event for cleaning, employees from other institutions come to help. At the same time, there are very often warning signs that people can see all the time (TE2)”. “Forest therapy sessions, cooperation with health institutions—development of therapy, promotion of physical activity, cognitive educational activities, attraction of foreign partners and specialists”, Education and awareness” (LE1). “Also, the recreational infrastructure of the forest is constantly being improved, the development of educational tourism is being developed, and opportunities for quiet rest in nature are being created (mushrooming, walking in the forest, relaxing by the water, recreational and educational trails for pedestrians and cyclists, etc.) (LFM3)”. “One of the main measures is ecological education and the development of citizens’ self-awareness (LSE2)”.*

In line with the answers given to the question about what should be done to protect the environment and forest areas, the view that legal improvements should be made in Turkey comes to the fore (Table 6). The statements of the experts participating in the research from Turkey are as follows:

**Table 6:** Experts’ opinions on protected clean and safe environment and rights to a clean and safe environment in forest areas.

The measures taken for environmental protection concerns to organized events.	
Experts in Turkey	Stricter regulations, collaborations.
Experts in Lithuania	More staff, water pollution, control of treatment facilities, organize educational tourism, education, prevention.

*“We need deterrent sanctions because people act recklessly. For example, they should know what can happen if they damage a tree. Another issue is that tobacco and tobacco products should be banned (TFM1)”. Laws or regulations should be more comprehensive. As someone who works here, protecting nature should not be my only duty. Everyone should be made to take responsibility. Otherwise, it is insufficient. In my opinion, laws and regulations need to be more comprehensive and dissuasive (TE2)”. “Laws must be clear and deterrent. People should obey the law in this regard. Otherwise, it is very difficult to control when there is no sanction” (TSE1).*

According to the opinions of experts from Lithuania, the view that training activities should be increased comes to the fore (Table 6). The statements of the experts participating in the research from Lithuania are as follows:

*“The country's priority and Lithuania's business card is forest tourism. The image of Lithuania is a climate resort country where recreation takes place all year round” (LFM1). “Organize educational tourism, education, prevention, education, limit illegal constructions, solve the problem of access to water, tighten the order regarding littering, water pollution, control of treatment facilities” (LE2). “First of all, public education and awareness raising. The biggest problems of recreational forests arise from people's non-observance of elementary rules. Secondly, efforts must be made to preserve the landscape and biological diversity of Lithuania, to promote the restoration of damaged natural elements, to ensure the rational use of the landscape and biological diversity” (LE2)”. “The visit of individuals to both state and private forests is free to promote people's citizenship, carry out prevention and promote awareness (LSE2)”.*

## 5 Discussion

Nature-based recreation activities are the most demanded outdoor recreation activities of today's city people to relieve fatigue and stress. As a result of these activities, people prefer areas such as parks and forests to get fresh air by staying alone with nature. Therefore, today's recreational activities in forest areas are very important for the development of health and social networks. To meet the increasing demand for this activity among citizens, various events and outdoor activities are organized. This research focuses on the relationship of forest areas open to recreational activities in Turkey and Lithuania with access to clean environment and environmental sustainability. In this context, the findings related to the recreational activities organized in forest areas in both countries, the environmental effects of these activities and the measures to be taken were evaluated.

Within the scope of the research, it is seen that different activities and organizations are organized in Lithuania compared to Turkey, in line with the answers given to the question of which recreational activities are organized in forest areas open to recreation. While the events organized in Turkey are limited to running, walking, and cycling. In addition to these, it can be said that activities in many different groups such as fishing, bird watching, horse riding, training camps are organized in Lithuania.

According to the areas where they are organized, recreation activities are evaluated under two headings as outdoor recreation activities and indoor recreation activities (78). Activities held in open areas take place in very different environments, including forests, lakes, rivers, national parks, and mountainous areas (79). For this, national and inter-national institutions, organizations and companies pay more and more attention to the negative effects they cause on the environment and try to adopt sustainable management practices in order to improve or reduce these negative effects (80–82). As people turn to nature, recreational activities that can be done in nature increase, diversify and encourage, and this allows more recreational activities to take place in nature (83, 84). In woodlands, hiking, bird watching, grass skiing, mountaineering, camping, mountain biking, rock, running, hiking, rock climbing, wildlife watching, horseback riding, navigation, cycling, summit walking, shooting, safari, hunting and backpacking activities such as traveling with a backpack are organized (85–87).

Analysis of the answers given to the question regarding the problems that emerged during the activities organized showed that the activities organized in the forest area mainly cause the problem of garbage. Interviewers from both countries indicated this common problem. It was also highlighted water pollution, illegal fishing and camping activities were carried out in Lithuania. However, it is understood that the participants in Lithuania, whose recreation tools and equipment were also damaged, acted insensitively on this issue.

The attitudes and behaviors of individuals and societies towards the environment can reduce the damage to the environment and a new ecological paradigm can be created in creating a livable environment. Thus, threats to the environment can be reduced in the relationship between humans and the environment. Destruction of nature can be pre-vented by protecting and developing open space recreation areas such as potential sea, lake, mountain, parks and gardens that are used for recreational purposes (88). Participation in recreational activities, especially outdoor recreation activities, can increase awareness in terms of environmental awareness and understanding of the deterioration in the ecological balance of the new ecological paradigm. For this reason, it is important to determine the environmental attitude of those participating in recreational activities regarding the new ecological environmental paradigm (89). According to the research, it has been determined that each recreational activity can cause different degrees of negative effects on the environment (90, 91).

In a study conducted by Mansuroğlu & Dağ (92), it was concluded that a high amount of garbage production occurs during recreational activities, and therefore the ecosystem in the region is greatly damaged. More than four billion tons of waste is produced every year and recycling of these wastes is getting harder day by day (93). Especially recreational activities, increase in artificial environment, change in feeding habits of wild animals, garbage problem, noise, solid waste etc. leads to negative effects such as (6). Since recreational areas are located in natural environments, waste generation can be the most visible negative impact. According to the results obtained, 87.5% of the recreation areas carry out waste collection on site, while only 25% record the waste they produce. In 31.3% of these facilities, the amount of waste varies between 1,000–2,500 kg per month, while the rest generate more than 2,500–20,000 kg of waste per month (94). In addition, many major and irreversible problems may be encountered in order to provide short-term economic benefits in natural landscape areas together with recreational activities (95, 96). However, recreational activities; air pollution, erosion, loss of biodiversity, increase of viruses and bacteria in the area, reduction of natural land-scape; On the other hand, the increase in the artificial environment, the change in the feeding habits of wild animals, the problem of garbage, noise, solid waste, etc. leads to negative effects such as (6, 97). In order to prevent this situation, it is of great importance for visitors to pay attention to environmental cleanliness, especially in the accommodation/break areas and picnic areas designated for activities that can last for a few days such as camping, mountaineering, trekking (92).

The reviewers' answers to the question regarding the legal regulation of the protection of forest areas revealed that the protection of forest areas open for recreation is legally regulated in both Turkey and Lithuania. In 78 out of 92 nations in the world, environmental laws were strengthened after the right to a healthy environment gained constitutional status. Laws were amended to specifically focus on environmental rights, as well as access to environmental information, participation in decision making, and access to justice (98). Of course, legal regulations are not the only factor contributing to the development and improvement of environmental laws. For example, the accession processes of the countries in Eastern Europe to the European Union have had a great impact on the formation of the environmental legislation of the countries. It is also known that these countries receive support for forming public opinion and legislative support from other jurisdictions during the membership process (99).

Forests, which are one of the most important elements of the environment, have a vital importance for people with their ecological and social benefits. Forests, which are the determinants of many vital issues such as clean air, climate change, water flow, soil fertility, and wildlife, provide economic benefits such as raw materials, energy resources and land, as well as ecological benefits (100). Considering that these benefits increase the quality of human life, it is very important to protect forest areas and all other natural resources and to manage them with a sustainable approach. With the spread of conservation awareness around the world, many developed and developing countries, within their borders; It can ensure the sustainability of both human life and nature by protecting animal and plant existence and habitats within the scope of certain criteria (101).

The results of experts answers analysis to the question regarding the measures taken during recreational activities are similar. Accordingly, it is seen that similar measures have been taken in both Turkey and Lithuania, such as raising education and awareness, increasing more employees and volunteering activities.

Recreational activities in nature can directly or indirectly affect the natural environment. It is to prevent damage to the natural environment in the area where the activities are carried out by ensuring that individuals who actively or passively participate in recreational activities while planning recreational activities in nature benefit from the opportunities of nature. With the understanding of sustainable environment, the natural environment is largely. At the same time, it can increase the use potential of natural areas and ensure that they reach future generations. Considering the sustainable environment approach, we can list the elements to be considered while planning recreational activities in nature as follows (102). Many different security and safety measures are taken for organized events in forested areas, as more people gather in forested areas. This is necessary for the sustainability of activities and forest areas as well. For this reason, the basic principles are to observe the balance of conservation and use in the area, to meet the needs and demands of the participants at an optimal level, to increase people's awareness of nature, to make area arrangements, to ensure that the tools to be used in the activities are compatible with the natural texture and character. However, in order to protect the existing areas and ensure continuity, the people of the region and the relevant institutions should work together and raise awareness (103). It is possible to prevent environmental problems caused by organized events and to take some basic precautions. Some of the measures that can be taken in this regard are the features that can be applied by changing them according to the economic, social, political, ecological and even cultural characteristics of the countries. Some are the measures that every country must implement (104).

Under the opinion of the interviewees, legal improvements is required in Turkey, and in Lithuania—greater attention should be paid to ecological education. In addition, it can be said that awareness and more information studies should be carried out for both countries.

Today, not only the economic and sociocultural benefits of recreational activities, but also their contribution to sustainability is becoming an important issue (105). It is known that there is a need for efficient planning that will guide recreational areas with a political understanding (23). It is necessary for the administrations to maintain the balance in a way that will both facilitate the access of people to the recreational areas in the forest and prevent the degradation of natural life (30). The protection, regulation and protection of the environment, which is of great importance in outdoor recreation activities, is a very important issue. Because, in this way, more recreational activity areas can be provided for cities and people can be allowed to lead a healthy life in a sustainable environment. In line with these purposes, practices such as the protection of wild nature, the regulation of certain regions for individuals participating in recreational activities, together with legal regulations, can be made (106). Informing people about environmental management can make a utilitarian and ecological understanding sustainable (107). Due to the activities held in forest areas, the natural environment and forests are seriously damaged. In addition, wastes such as garbage and plastic caused by the participants are also a great threat to the ecosystem. The main reason for this situation is that societies do not have a conscious consumer profile (19).

Recreational activities and mobility put great pressures on eco-logical systems. It has become imperative to create new policies and protection measures in order not to endanger the lives of living creatures in nature, not to threaten the continuity of the generations of animals, and to protect the sustainability of the resources we use as a recreational resource (108). The activities carried out in the natural environment should be examined in a multifaceted way and a detailed activity plan should be made. Thus, it may be possible to harm nature less. Therefore, the fact that the people participating in the events act with an environmentally friendly understanding is the main element of the event and environmental sustainability. For this, individuals should prefer environmentally friendly products in the events they attend, and act by considering environmental degradation in all activities (109). In addition, the sustainable development of recreational areas depends on the preservation of the environmental characteristics of the area and its planned development (110). The planet we live in has been facing increasing environmental problems in recent years. In the fight against these problems, environ-mental participation during recreational activities and establishing a connection with nature can contribute to this process (111). However, in order to better understand the value of the natural environment and to ensure the sustainability of the environment, the awareness of individuals regarding the use of the natural environment in their participation in recreational activities should be increased (106).

## 6 Conclusion

Recreation activities in forests provide a unique opportunity to connect with nature and enjoy the great outdoors. Whether it be hiking through picturesque landscapes, camping under the stars, or fishing in a pristine stream, forests offer a peaceful and natural environment to engage in these activities. They also provide a break from the hustle and bustle of modern life and allow individuals to disconnect and recharge.

The environment provides a habitat for plants and animals, and preserving these natural resources is essential for maintaining the biodiversity of the area. Recreationists are drawn to areas with abundant wildlife and plant life, and protecting the environment ensures that these resources remain available for recreational purposes. The environment provides the resources that make recreation possible, and protecting these resources is essential for ensuring that recreation can continue into the future. By promoting sustainable practices and responsible use of natural resources, we can help to preserve the environment and ensure that it remains available for recreation for years to come. Protecting the environment is crucial for providing opportunities for recreation. By pre-serving natural resources, maintaining clean air and water, reducing human impact, and promoting sustainability, we can ensure that these areas remain available for recreation for generations to come. It is very important to protect the ecosystem during recreational activities in forest areas and to develop sensitive behaviors to prevent damage to forest areas. Choosing low-impact activities in terms of the environment and complying with waste practices and rules can contribute to the protection of forest areas. By promoting responsible recreation, the natural environment can be protected for current and future generations.

In the research, recreational activities organized in forest areas in the cities deter-mined in Turkey and Lithuania, the environmental effects of these activities and the precautions to be taken were determined. An evaluation has been made in terms of both countries. As a result of the interviews conducted within the scope of the research, the following conclusions were reached:

• While the recreational activities organized in Turkey are limited to running, walking and cycling; In addition to these, it is understood that activities in many different groups such as fishing, bird watching, horse riding, training camps are organized in Lithuania. While the recreational activities organized in Turkey are limited to running, walking and cycling; In addition to these, it is understood that activities in many different groups such as fishing, bird watching, horse riding, training camps are organized in Lithuania.
• It is seen that the main problem that stands out about the events held in both countries is the garbage and waste produced by people.
• It is understood that legal arrangements have been made in both countries with the aim of protecting and sustaining forest areas and special laws and regulations have been prepared on this issue.
• It is observed that similar measures have been taken in both countries during the recreational activities, such as increasing education and awareness, in-creasing more employees and volunteering activities.
• While the opinion that legal improvements should be made in Turkey for the protection of the environment and forested areas, it is seen that measures related to education are prominent in Lithuania. However, the opinion that awareness and more information studies should be carried out for both countries comes to the fore.

In a period where the importance of activities organized in forest areas is increasing, it is very important to ensure the sustainability of both the activities and the forest areas. Summarizing the research results, we can note that it is necessary to take appropriate measures to increase people's awareness and improve legal regulation. In addition, in the case of Turkey, it is recommended to organize modern and suitable for all social groups activities to attract more participants. It can be said that necessary measures should be taken, awareness of people should be increased, and legal regulations should be improved. In addition, the preparation of different and richer event contents in Turkey can enable more people to participate. This research is limited to two countries, but the results can be considered as an opportunity to carry out more comprehensive studies. This study, which was carried out in Turkey and Lithuania, can be considered as the first study in terms of revealing the relationship between recreation and environmental sciences. In addition, it is aimed to contribute to the field as a unique study in terms of the way two different countries are handled and evaluated in terms of population and surface area. It can also be seen as an opportunity to carry out different studies with the participation of more than two countries in the future with more comprehensive and large masses.

## Data Availability

The original contributions presented in the study are included in the article/Supplementary Material, further inquiries can be directed to the corresponding author.
